# Psychometric Properties of the Parent Versions of the Japanese Versions of the Strength and Difficulties Questionnaire: A study on Health Checkups for 5-Year-Old Children in Japan

**DOI:** 10.1192/j.eurpsy.2023.475

**Published:** 2023-07-19

**Authors:** K. Yokoyama, K. Nomura

**Affiliations:** Nagoya University, Nagoya, Japan

## Abstract

**Introduction:**

In Japan, the effectiveness of health checkups for children aged 5 years has attracted attention as the basis for a support system for early detection and support of children with developmental disabilities. However, these have not yet become statutory health checkups, and their assessment has not been standardized. This study employed the Strength and Difficulties Questionnaire (SDQ) in examining the health of a 5-year-old child. This study aims to demonstrate the scores’ distribution and obtain the standard land and cutoff values of the SDQ.

**Objectives:**

From 2010 to 2012, children reaching 5 years of age in Kanie-cho, Aichi Prefecture, Japan, underwent a health checkup. Of the 888 children for whom parental consent was obtained, 884 responses without missing values (453 boys: 51.2%, 430 girls: 48.6%) were included in the analysis.

**Methods:**

SDQ and medical questionnaires for 5-year-old children were sent to the parents with a formal request for approval for the child to participate in the study. Further, the parents were asked to respond to the questionnaire. The Ethics Committee of the Graduate School of Education and Developmental Sciences, Nagoya University approved this study (No.298).

**Results:**

A confirmatory factor analysis using the maximum likelihood method revealed a factor structure almost identical to that of Goodman (JCPP 1997; 38 581-586). Nonetheless, items 3, 10, 11, 13, 14, and 22 showed high loadings on factors different from those in the original version. Similar to the original version, we set our criteria so that Some and High Needs would be approximately 10% each. Furthermore, we calculated the percentage of children who fell into these categories and found that the scores that fell into the Need category differed from those in the previous study(Table 1).

**Image:**

**Figure:**
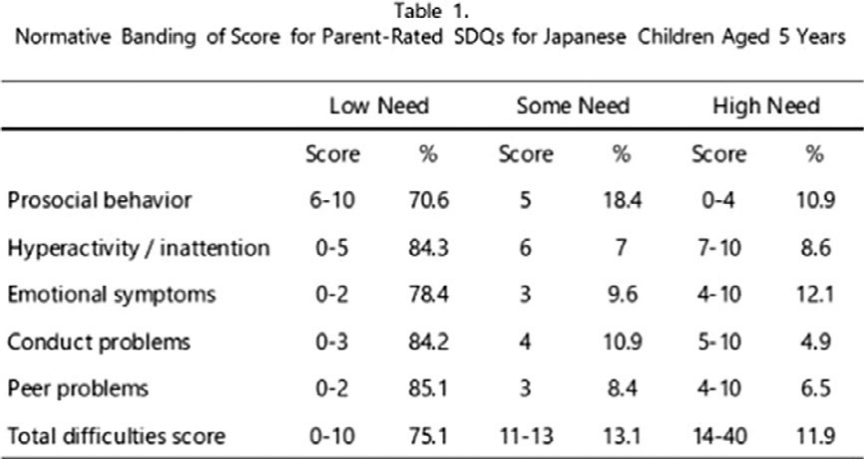

**Conclusions:**

Although the five-factor structure of the SDQ was generally accepted, as pointed out in previous studies (Matsuishi *et al*. Brain Dev 2008;30 410-415 : Iida *et al*. 2014; 33-41), differences in expression between English and Japanese and cultural differences may have influenced the results. Therefore, it is necessary to be careful in interpreting the results. Additionally, the differences in the Need classification indicated that the difficulty level tends to be slightly lower in the 5-year-old children’s health checkups and that boys may be more likely to recognize the “Hyperactivity/inattention” problem. Based on these results, it is necessary to consider more effective ways of using the SDQ in 5-year-old children’s health checkups, such as evaluating the SDQ scores in combination with the actual condition of the children at the time of the checkup.

**Disclosure of Interest:**

None Declared

